# Increased risk of attention-deficit/hyperactivity disorder in adolescents with high salivary levels of copper, manganese, and zinc

**DOI:** 10.1007/s00787-024-02381-2

**Published:** 2024-02-14

**Authors:** D’Artagnan M. Robinson, Karen L. Edwards, Michael T. Willoughby, Katrina R. Hamilton, Clancy B. Blair, Douglas A. Granger, Elizabeth A. Thomas

**Affiliations:** 1 Department of Epidemiology and Biostatistics, University of California Irvine, Irvine, CA USA; 2 RTI International, Research Triangle Park, NC USA; 3 Department of Psychiatry and Behavioral Sciences, John Hopkins University, School of Medicine, Baltimore, MD USA; 4 Institute for Interdisciplinary Salivary Bioscience Research, University of California Irvine, Irvine, CA USA; 5 Department of Population Health, New York University Grossman School of Medicine, New York, NY USA; 6 Johns Hopkins University School of Nursing, Bloomberg School of Public Health, and School of Medicine, Baltimore, MD USA; 7 Department of Neurobiology and Behavior, University of California, Irvine, Irvine, CA USA

**Keywords:** Attention-deficit and hyperactivity disorder, Saliva, Metals, Subtype

## Abstract

Exposure to toxic heavy metals has been associated with the development of attention-deficit/hyperactivity disorder (ADHD). However, fewer studies have examined the associations between abnormal levels of essential trace metals and ADHD, and none have done so using saliva. We investigated whether salivary metals were associated with ADHD in adolescents aged 12 from the Family Life Project (FLP) using a nested case–control study design that included 110 adolescents who met diagnostic criteria for inattentive (ADHD-I), hyperactive-impulsive (ADHD-H), or combined type ADHD (ADHD-C) (cases) and 173 children who did not (controls). We used inductively coupled plasma optical emission spectrophotometry to measure chromium, copper, manganese, and zinc in saliva samples. We employed logistic regression models to examine associations between quartile levels of individual metals and ADHD outcomes by subtype. Salivary copper levels were significantly associated with increased odds of any ADHD diagnosis (OR = 3.31, 95% CI: 1.08–10.12; *p* = 0.04) and with increased odds of ADHD-C diagnosis (OR = 8.44, 95% CI: 1.58–45.12; *p* = 0.01). Salivary zinc levels were significantly associated with increased odds of ADHD-C diagnosis (OR = 4.06, 95% CI: 1.21–13.69; *p* = 0.02). Salivary manganese levels were also significantly associated with increased odds of ADHD-C diagnosis (OR = 5.43, 95% CI: 1.08–27.27, *p* = 0.04). This is the first study using saliva to assess metal exposure and provide a potential link between salivary levels of copper, manganese, and zinc and ADHD diagnoses in adolescents. Public health interventions focused on metal exposures might reduce ADHD incidence in low-income, minority communities.

**Supplementary Information:**

The online version contains supplementary material available at 10.1007/s00787-024-02381-2.

## Introduction

Attention-deficit/hyperactivity disorder (ADHD) is a neurodevelopmental disorder that is characterized by age-inappropriate, persistent patterns of excessive inattentiveness, hyperactivity, and impulsivity that causes impairment across a variety of settings (i.e., school, work, home, etc.) [1]. ADHD is a complex disorder with both genetic and environmental factors contributing to its susceptibility and risk [2, 3]. Current prevalence data report that 6.1 million children in the United States suffer from ADHD, while 2019 global prevalence data suggests 84.7 million people are affected worldwide [4, 5]. Despite ADHD standing as the most diagnosed mental disorder of childhood and adolescence, little is known about the disease etiology, particularly regarding the environmental impacts of metal exposures on ADHD symptoms and diagnosis [6].

The neurotoxic effects of heavy metals, such as mercury and lead, are well documented [7–11], but less is understood about the potential impacts to ADHD risk conferred by other metals, some of which are required in the body at trace amounts (<0.01%) but can be toxic at sufficiently high levels. These trace metals, such as zinc, manganese, copper, iron, and magnesium, are essential for numerous biological and chemical processes, including regulating cellular homeostasis, acting as cofactors for many enzymes, and serving as antioxidant molecules [12]. Current epidemiologic investigations into the associations between bodily concentrations of trace metals and ADHD remain inconclusive, but the emerging body of evidence suggests that either heightened or insufficient exposure to several essential metals can influence the odds of ADHD diagnosis and/or symptoms [13–18]. Most of these past studies have relied on metals quantified in blood, urine, or hair samples; however, no studies have attempted to investigate levels of metals in saliva in relation to behavioral function in children. Although some studies have revealed significant correlations between serum and saliva for certain metals, such as manganese and copper [2, 3], levels of other metals, such as zinc, cadmium, or lead, in saliva were not found to be significantly correlated with blood levels [19–21].

Saliva has many advantages as an alternative biofluid to blood, as it non-invasive and easy to collect, does not require trained personnel or immediate processing, and samples can be collected in any setting. Saliva also allows for regular or routine sampling over time, which could be important for individuals with high risk for behavioral or neurological disorders. Yet, despite emerging studies implicating saliva as a reliable indicator of environmental exposure to metals [21], only one study has examined salivary metal levels in a pediatric population of ADHD cases, and that study exclusively investigated the impact of salivary mercury on ADHD risk [22]. The United States Environmental Protection Agency has not developed regular standards for appropriate levels of metals; hence, the dearth of research on this topic could be due to, in part, the fact that no permissible limits or guidelines yet exist for salivary metal levels [23].

Since saliva biomarker exposure assessment might prove useful as a non-invasive, cost-effective tool for evaluating ADHD risk in adolescents, the goal of this study was to investigate the associations between salivary metals on ADHD risk and also consider different subtypes of the disorder.

## Methods

Participants: This study was reviewed and approved by the Office of Human Research Ethics at the University of North Carolina (UNC IRB (#07-0646; #16-2751)) and involves subjects recruited as part of the Family Life Project (FLP), a prospective, population-based longitudinal study that began in 2003. This project consists of adolescents and their caregivers, followed from birth in predominantly low-income, non-metropolitan counties in Pennsylvania and in North Carolina. A detailed characterization of the sampling plan and study has been provided elsewhere [24]. This study uses data and specimens collected from home visits when adolescents were 12 years of age, with parents or primary caregivers providing consent. Family income-to-needs ratio was assessed at this visit using a parent/caregiver report of total household income and the number of adults and children in the household. The sample size of those that received home visits was *n* = 893; however, only *n* = 508 of those adolescents provided a saliva sample with a volume >1 mL, given that ~0.8 mL volume was required to run the metal panel quantification. Participants in this nested case–control study were selected from the 508 adolescents that provided adequate saliva samples and adequate information for classification according to the ADHD diagnostic criteria below, resulting in total sample size of this nested case–control study is *n* = 283.

Diagnostic procedures: Trained research assistants administered the *Diagnostic Interview Schedule for Children* (DISC-IV), a fully structured diagnostic instrument that included criteria according to the *Diagnostic and Statistical Manual of Mental Disorders* (4th ed.; DSM-IV; American Psychiatric Association, 2013), to the primary caregiver of target adolescents. Scoring adheres to the DSM-IV and includes past month (embedded current) and past year diagnoses, as well as impairment scores, based on six impairment domains and three levels of severity. The predominantly Inattentive Type of ADHD (ADHD-I) included individuals with six or more symptoms of inattention and fewer than six symptoms of hyperactivity-impulsivity; the predominantly Hyperactive-Impulsive Type (ADHD-H) included individuals with six or more symptoms of hyperactivity-impulsivity and fewer than six symptoms of inattention; the Combined Type (ADHD-C) is defined by six or more symptoms on both dimensions. Non-ADHD controls are defined as adolescents that displayed either no ADHD symptoms or only one symptom of either ADHD-I or ADHD-H type to avoid potential misclassification bias.

Saliva sample collection: Unstimulated whole saliva samples were collected from adolescents using the passive drool method during the 12-year visit to participants’ homes [25]. At the time of collection, samples were frozen at −20 °C and then transferred to the Institute for Interdisciplinary Salivary Bioscience Research at the University of California, Irvine, for archiving at −80 °C until used. At the time of use, saliva samples were thawed and centrifuged (5000*g*; 10 min; 4 °C) to remove insoluble material and cellular debris. Supernatants were collected and used for other assays separate from the current study. For the current study, samples with at least 0.8 mL of saliva remaining were used, to allow for enough volume to run the metal analysis.

Salivary metal analysis: Levels of metals in saliva were measured using Inductively Coupled Plasma Optical Emission Spectrometry (ICP-OES) (Avio200; Perkin Elmer, Waltham, MA, USA) using the radial viewing window [26]. All glassware was treated with 10% (volume/volume; v/v) HNO3 before use and then rinsed with deionized water. Samples were diluted 1:7 in 2% HNO3. Metal output was normalized to the internal standard, 0.1% Yttrium, and compared to standard curves generated for each metal, which were prepared from stock solutions of 1000 ppm. The ICP-OES was operated with argon carrier flow rate of 0.5 L/min, plasma gas flow rate of 15 L/min, sample flow and elusion rate of 1.51 L/min, and peristaltic pump speed of 100 rpm. The following suitable wavelengths were selected for each element: chromium (267.716), copper (327.393), manganese (257.610), and zinc (206.200). The analytical detection limits for all biomarkers are based on repeated measurements of procedural blanks on four separate analysis days. The lowest level of detection for each metal was the following: chromium (0.14 µg/L), copper (0.45 µg/L), manganese (0.07 µg/L), and zinc (0.18 µg/L).

Statistical analysis: SAS® Version 9.4 (Cary, NC) and IBM® SPSS® Statistics (version 25 for Windows; BM Corp., NY, USA) were used for all data analysis. Salivary metal data were first tested for normal distribution using QQ-plots and histograms and for potential outliers using boxplots. Metal data were not normally distributed, nor did log-transformation improve normality. Further testing of potential outliers utilized statistical measures of influence (i.e., Pearson residuals, delta chi-squares, delta deviances, delta-betas, leverage diagnostics, predicted probability diagnostics, C and CBAR) to determine if individual observations should be deleted from final regression models. Potentially influential outlier observations were deleted in a piecewise manner to evaluate changes in odds ratio (OR) estimates. The deletion of potential outliers did not result in significant changes to OR estimates, and therefore, no individual observations were omitted from the final models. Associations between salivary metal levels and participant characteristics were determined using Kruskal–Wallis and *χ*^2^ tests. Comparison of participant characteristics by case–control status for all subtypes were performed using *χ*^2^ and Mann–Whitney tests. Differences in metal concentrations by ADHD diagnosis status were determined by performing Mann–Whitney tests.

To estimate the associations between ADHD diagnostic status and salivary metal levels, we calculated odds ratios (ORs) with 95% confidence intervals (CIs) by performing a series of logistic regression analyses, with consideration of specific ADHD diagnostic subtypes. However, the low sample size (*n* = 12) of the ADHD-H case group did not allow for logistic regression analyses to be performed separately for this diagnostic subtype. Therefore, logistic regression results are presented only for the following three case subtypes: (1) total ADHD cases; (2) ADHD-I; (3) ADHD-C. In each logistic regression analysis, binary diagnostic variables (where ‘0’ indicates control, ‘1’ indicates case) were regressed against salivary metal level quartiles. This series of logistic regression analyses examines the associations for the three aforementioned ADHD outcomes with all four metals of interest, separately. Salivary metal levels were examined as categorical variables based on quartile levels, whereby the distribution of salivary metal concentrations within the entire analytic sample was used to determine quartile boundaries. Specifically, quartiles were created by dividing the salivary metal data into four groups, with each quartile group representing 25% of the total distribution. We utilized the first quartile group as the reference group across all logistic regression analyses. This approach allows us to compare the odds of each ADHD diagnostic subtype status across different levels of salivary metal concentrations, using the first quartile group for comparison. For all analyses, *α* was set to a significance level of 0.05.

We evaluated for potential confounding by sex, race, state of residence, body mass index, and income-to-needs ratio through regression model adjustment and subsequently assessing the impact of covariate adjustment on OR estimates. Logistic regression models were adjusted for sex, race, state of residence, body mass index, and income-to-needs-ratio when indicated, as determined by sizable changes in OR estimates when variables were included in the crude model.

## Results

### Salivary metals and adolescent/family characteristics

Table 1 reports adolescent characteristics for each case group and the control group. ADHD cases, across all groups, and the control group, differed by sex, with case groups consisting of a greater proportion of males. ADHD cases, across all case groups, and the control group did not significantly differ by race, state of residence, BMI, or income-to-needs ratio (Table 1).

**Table 1:** Characteristics of FLP subjects by case–control status

Variable	Mean ± SD or *N* %				
	Controls (*N* = 173)	ADHD—total (*N* = 110)	ADHD-I (*N* = 65)	ADHD-H (*N* = 12)	ADHD-C (*N* = 33)
Age (years)	13.20 ± 0.60	13.13 ± 0.50	13.17 ± 0.56	13.06 ± 0.44	13.08 ± 0.39
Sex					
*Male*	84 (48.5%)	78 (70.9%)	45 (69.2%)	4 (33.3%)	25 (75.8%)
*Female*	89 (51.5%)	32 (29.1%)	20 (30.8%)	8 (66.7%)	8 (24.2%)
Race					
*Non-black*	102 (59.0%)	58 (52.7%)	36 (55.4%)	6 (50.0%)	16 (48.5%)
*Black*	71 (41.0%)	52 (47.3%)	29 (44.6%)	6 (50.0%)	17 (51.5%)
Body Mass Index	23.28 ± 6.21	24.06 ± 6.34	25.07 ± 6.98	23.46 ± 4.98	22.28 ± 5.05
Income-to-needs ratio	2.22 ± 1.94	1.72 ± 1.53	1.93 ± 1.77	1.69 ± 1.26	1.29 ± 0.92
Area of residence					
*North Carolina*	96 (55.5%)	63 (57.3%)	39 (60.0%)	7 (58.3%)	17 (51.5%)
*Pennsylvania*	77 (44.5%)	47 (42.7%)	26 (40.0%)	5 (41.7%)	16 (48.5%)

*ADHD-I* ADHD inattentive subtype, *ADHD-H* ADHD hyperactive/impulsive subtype, *ADHD-C* ADHD combined subtype

The median levels and ranges for each metal in saliva across case and controls groups are shown in Table 2. A comparison of the median level of chromium in the controls (5.33 µg/L) with the median level of the ADHD-H case group (7.20 µg/L) highlights a significant difference between the two groups for this metal. For the ADHD-C group, significantly higher median levels compared to controls were observed for both copper (8.46 vs. 4/80 µg/L) and zinc (34.7 vs. 22.67 µg/L). No other differences in median salivary metals were observed across the diagnostic groups (Table 2).

**Table 2:** Case–control comparison of median salivary metal levels

Metal	Medium (range)				
	Controls (µg/L)	ADHD—total (µg/L)	ADHD-I (µg/L)	ADHD-H (µg/L)	ADHD-C (µg/L)
Chromium	5.33 (1.67–11.66) *N* = 173	5.19 (1.25–13.45) *N* = 110	4.88 (1.25–9.68) *N* = 65	7.20^*^ (4.05–9.11) *N* = 12	5.27 (2.20–13.45) *N* = 33
Copper	4.80 (2.24–48.07) *N* = 78	5.79 (2.35–434.22) *N* = 58	5.47 (2.39–434.22) *N* = 32	11.36 (4.26–16.29) *N* = 4	8.46^*^ (2.35–29.46) *N* = 22
Manganese	1.86 (0.40–15.42) *N* = 78	2.19 (0.47–19.09) *N* = 58	1.66 (0.47–18.06) *N* = 32	3.01 (0.53–7.18) *N* = 4	2.62 (0.50–19.09) *N* = 22
Zinc	22.67 (6.23–457.92) *N* = 173	26.05 (1.12–236.03) *N* = 110	20.56 (1.12–178.50) *N* = 65	33.22 (9.60–66.09) *N* = 12	34.70^*^ (10.23–236.03) *N* = 33

An asterisk (^*^) indicates a *p* value <0.05 for Mann–Whitney *U* test comparing median levels between cases and controls

A result in bold font indicates a *p* value <0.01 for Mann–Whitney *U* test comparing median levels between cases and control

The differences in *N* for certain metals are due to samples that fell below the detection limits of the instrument for that metal

### Salivary metals and ADHD risk

We next investigated the relationship between salivary metals and ADHD diagnosis in a nested case–control design. Salivary metals were compared according to quartiles (Supplementary Table 1), including all cases and controls, with the first quartile serving as the reference. Table 3 presents the results of the logistic regression analyses that examined total ADHD cases as the main outcome. Salivary copper showed the strongest association with ADHD diagnosis odds, as adolescents with salivary copper levels in the second, third, and fourth quartiles showed 1.84 (95% CI: 0.61–5.56), 2.99 (95% CI: 0.98–9.13) and 3.31 (95% CI: 1.08–10.12) times greater odds, respectively, of any type ADHD compared to adolescents with copper levels in the first (reference) quartile. Assessing only the ADHD-I subtype (Table 4), no salivary metal was associated with increased or decreased odds of an ADHD-I diagnosis.

**Table 3:** Odd ratios (OR) and 95% confidence intervals (CI) for total ADHD cases in association with salivary metals

	*N* (cases)	*N* (controls)	Any Type ADHD—OR (95% CI)	*p* value
Chromium (Cr)^*^				
1st quartile	31	38	Reference	N/A
2nd quartile	25	46	0.67 (0.34, 1.32)	0.24
3rd quartile	22	49	0.55 (0.28, 1.10)	0.09
4th quartile	31	40	0.95 (0.49, 1.85)	0.88
Copper (Cu)^**^				
1st quartile	8	25	Reference	N/A
2nd quartile	15	19	1.84 (0.61, 5.56)	0.28
3rd quartile	16	18	2.99 (0.98, 9.13)	0.05
4th quartile	18	15	**3.31 (1.08, 10.12)**	**0.04**
Manganese (Mn)^*^				
1st quartile	17	16	Reference	N/A
2nd quartile	11	23	0.45 (0.17, 1.21)	0.11
3rd quartile	11	23	0.45 (0.17, 1.21)	0.11
4th quartile	19	15	1.19 (0.46, 3.12)	0.72
Zinc (Zn)^*^				
1st quartile	25	45	Reference	N/A
2nd quartile	25	45	1.00 (0.50, 2.00)	1
3rd quartile	26	45	1.04 (0.52, 2.07)	0.91
4th quartile	34	37	1.65 (0.84, 3.25)	0.14

An asterisk (^*^) indicates a univariate final model

Two asterisks (^**^) indicates the model is adjusted for sex

**Table 4:** Odd ratios (OR) and 95% confidence intervals (CI) for ADHD, inattentive subtype, in association with salivary metals

	*N* (cases)	*N* (controls)	Inattentive Type ADHD—OR & 95% CI	*p* value
Chromium (Cr)^*^				
1st quartile	22	38	Reference	N/A
2nd quartile	15	46	0.56 (0.26, 1.23)	0.15
3rd quartile	13	49	0.46 (0.21, 1.03)	0.06
4th quartile	14	40	0.60 (0.27, 1.35)	0.22
Copper (Cu)^**^				
1st quartile	6	25	Reference	N/A
2nd quartile	9	19	1.48 (0.42, 5.17)	0.54
3rd quartile	10	18	2.69 (0.76, 9.50)	0.13
4th quartile	6	15	1.53 (0.39, 6.00)	0.55
Manganese (Mn)^**^				
1st quartile	13	16	Reference	N/A
2nd quartile	5	23	0.31 (0.09, 1.11)	0.07
3rd quartile	6	23	0.39 (0.12, 1.32)	0.13
4th quartile	8	15	0.76 (0.23, 2.51)	0.65
Zinc (Zn)^***^				
1st quartile	19	45	Reference	N/A
2nd quartile	18	45	1.17 (0.53, 2.59)	0.71
3rd quartile	15	45	0.89 (0.40, 2.01)	0.79
4th quartile	13	37	0.91 (0.39, 2.11)	0.82

An asterisk (^*^) indicates a univariate final model

Two asterisks (^**^) indicates the model is adjusted for sex

Three asterisks (^***^) indicates the model is adjusted for BMI

**Fig. 1:**
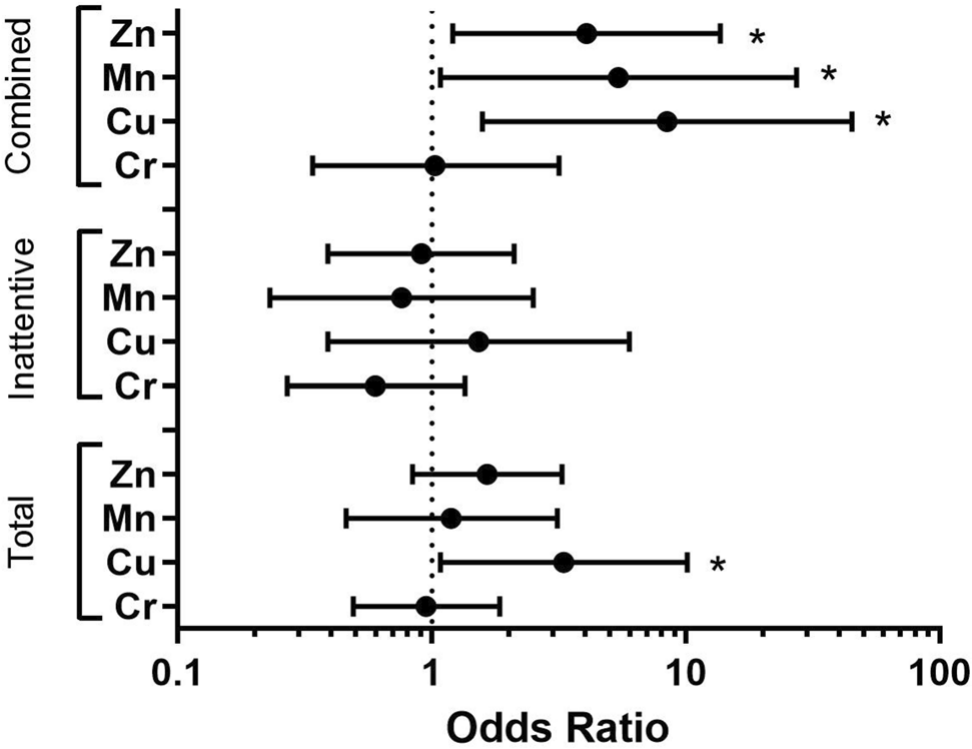
Summary of the odds ratios and 95% confidence intervals for ADHD in association with salivary metal levels

Finally, we assessed the association between salivary metals and adolescents with combined type ADHD (Table 5). The odds of combined type ADHD among adolescents in the fourth quartile of salivary copper exposure were 8.44 times that of the reference group (95% CI: 1.58–45.12). We also observed that the odds of combined type ADHD among adolescents in the fourth quartile of salivary manganese exposure were 5.43 times that of the reference group (95% CI: 1.08–27.27). Lastly, we found that the odds of combined type ADHD among adolescents in the fourth quartile of salivary zinc exposure were 4.06 times the odds of those in the first quartile reference group (95% CI: 1.21–13.69) (Table 5). Figure 1 summarizes the ORs of each salivary metal, comparing the fourth quartiles to the first quartile reference group.

**Table 5:** Odd ratios (OR) and 95% confidence intervals (CI) for ADHD combined type in association with salivary metals

	*N* (cases)	*N* (controls)	Combined Type ADHD—OR & 95% CI	*p* value
Chromium (Cr)^*^				
1st quartile	8	38	Reference	N/A
2nd quartile	7	46	0.62 (0.20, 1.96)	0.42
3rd quartile	8	49	0.68 (0.22, 2.10)	0.5
4th quartile	10	40	1.03 (0.34, 3.17)	0.95
Copper (Cu)^**^				
1st quartile	2	25	Reference	N/A
2nd quartile	5	19	4.32 (0.73, 25.74)	0.11
3rd quartile	5	18	2.91 (0.49, 17.19)	0.24
4th quartile	10	15	**8.44 (1.58, 45.12)**	**0.01**
Manganese (Mn)^*^				
1st quartile	3	16	Reference	N/A
2nd quartile	5	23	1.39 (0.28, 6.96)	0.69
3rd quartile	5	23	1.67 (0.33, 8.52)	0.54
4th quartile	9	15	**5.43 (1.08, 27.27)**	**0.04**
Zinc (Zn)^***^				
1st quartile	4	45	Reference	N/A
2nd quartile	4	45	0.77 (0.17, 3.45)	0.74
3rd quartile	10	45	2.17 (0.61, 7.64)	0.23
4th quartile	15	37	**4.06 (1.21, 13.69)**	**0.02**

An asterisk (^*^) indicates the model is adjusted for income-to-needs ratio and race

Two asterisks (^**^) indicates the model is adjusted for income-to-needs ratio

Three asterisks (^***^) indicates the model is adjusted for sex and BMI

## Discussion

The aim of this study was to examine the associations between essential trace metals quantified in saliva and ADHD outcomes in a subsample of adolescents from the FLP cohort at 12 years of age. This is the first study to our knowledge that utilizes salivary samples to measure levels of trace elements in adolescents with ADHD. Our results demonstrate significant associations between salivary copper, manganese and zinc levels with diagnoses of ADHD.

We found a significant near fourfold increased odds of a diagnosis of any type of ADHD for those adolescents with copper levels in the highest quartile compared to those with copper levels the lowest quartile, after adjustment for sex. These odds more than doubled (OR = 8.44, 95% CI: 1.58–45.12) when only the ADHD-C group was considered, after adjustment for income-to-needs ratio. Although the ADHD-C subtype was small (*n* = 33), this robust effect likely contributes to the increased odds when all subtypes are considered. These findings are consistent with previous studies using urine and hair samples that reported higher copper levels in ADHD cases compared to healthy controls [14, 27]. One particular study [27] showed that the odds ratios of ADHD increased approximately 12-fold for children with urinary copper in the fourth quartile compared to the lowest quartile of urinary copper. However, another study reported that deficiencies in copper levels as measured in hair samples were observed in ADHD cases compared to controls [28]. Yet, another study using whole blood samples did not detect significant differences between copper levels among ADHD cases and healthy controls [18]. One consideration is that the two above studies examined populations that were either entirely Egyptian or Han Chinese and included cases and controls aged 6–16 years of age, while participants in our study were approximately 50% Black and all approximately 12 years of age. Hence, factors related to genetics and age might account for some of the differences observed in previous studies.

While the role of copper in ADHD pathogenesis is still not explicitly understood, this metal is crucial for the catalysis of numerous enzymes (e.g. tyrosinase, dopamine hydroxylase, copper/zinc superoxide dismutase) linked to the developmental disorder [14]. Further, copper has been shown to cause damage in dopaminergic neurons throughout the substantia nigra by accelerating the production of free radicals and impairing antioxidant defenses [29]. Therefore, high levels of copper could lead to dopamine oxidation and copper-mediated neurotoxicity, offering one possible explanation for why increasing copper levels might be associated with increased risk for ADHD [14].

We also detected significant associations with zinc, mainly in relation to the ADHD-C subtype. Not only were median levels of salivary zinc higher in adolescents with ADHD-C, but further, we observed a statistically significant fourfold increase in the odds for ADHD-C diagnosis in adolescents with zinc levels in the highest quartile compared to the first quartile reference group, after adjustment for sex and BMI. These findings of increased odds for ADHD-C are consistent with one report showing higher levels of zinc in blood from children with ADHD compared to children without ADHD [30]. However, although ADHD subtypes were not explored, several other studies utilizing hair, urine, or blood samples found lower levels of zinc in children with ADHD compared to healthy controls [14–18]. Conflicting results might also be due to differences in control group definitions across studies, as previous research studies in this field have utilized healthy control groups, consisting of children confirmed as free of other severe medical disorders and not currently using any supplements or influential drugs [14–18]. Other studies have suggested that the copper/zinc ratio might be relevant to ADHD. For example, studies have shown that the serum copper/zinc ratio was correlated with teacher-rated scores for inattention, while another study reported higher serum copper/zinc ratios in ADHD children compared to the control group [15, 16].

Though median levels of manganese were not significantly different between cases and controls, we observed significantly increased odds of an ADHD-C diagnosis in adolescents with manganese levels in the highest quartile compared to the first quartile, after adjustment for race and income-to-needs ratio. This finding is consistent with other studies using blood and hair. For example, one study observed significantly higher levels of blood manganese in children with ADHD compared to controls [30], while another study found that children with hair manganese levels >3 μg/g had significantly higher scores on the ADHD Index subscale of the Revised Conner’s Teachers Rating Scale, compared to children with low levels of hair manganese [31]. Further, a recent meta-analysis of four case–control studies also observed significantly greater peripheral manganese levels in ADHD cases compared to controls [32].

Another study investigated manganese exposure based on levels found in drinking water. This study found that manganese in drinking water was associated with an increased hazards of the ADHD-I subtype, though not with the ADHD-C subtype [33]. These studies were carried out in an entirely Danish population, whereby our study consisted of ~50% Black children; hence, the difference in biofluid used to measure manganese exposure and the difference in racial composition of the cohort could explain why our study detected increased odds of ADHD-C, rather than ADHD-I. Other research studies have highlighted that blood and urinary manganese levels differed across race and were substantially higher among minority participants compared to non-Hispanic whites, as well as higher among residential areas with higher financial strain [34, 35]. These studies support adjustment for race and income-to-needs ratio when assessing the association between salivary manganese and ADHD diagnoses. Despite consistent findings, more studies are needed measuring manganese via saliva to assess for ADHD outcomes. While two previous studies have collected salivary manganese measures, neither study focused on ADHD outcomes [36, 37].

The role of manganese in ADHD development is still being understood, but research highlights that, although this metal is beneficial for its antioxidative properties at low concentrations, manganese is neurotoxic at high concentrations [38]. At high levels, manganese has been shown to accumulate in dopaminergic neurons in basal ganglia nuclei, which could affect dopamine neurotransmission [38]. Therefore, high levels of manganese exposure could lead to altered dopaminergic activity, which could explain why increasing levels of salivary manganese could be associated with increased odds of ADHD diagnosis [38].

Levels of trace metals in the body can be affected by both genetic and environmental factors. Environmental factors include geographical location, metal contamination in the soil, water or air, and socio-economic factors related to diet, dietary supplements, oral health, obesity, or smoking [39, 40]. Our previous studies have found correlations between salivary levels of copper, manganese and zinc and the nicotine metabolite, cotinine, in a younger subgroup (7 years) of children from the FLP cohort [41] suggesting that environmental tobacco smoke might represent a source of metal exposure. Additionally, a recent study characterized different sources of environmental exposures to metals and evaluated the ability of different biofluids to reflect these exposures [22]. Results showed that levels of chromium, copper, manganese and lead in soil, dust and air could be differentially revealed in blood, hair, nails, and saliva [22]. Further, the state of oral health in children might affect metal levels in saliva. Previous studies have investigated relationships between salivary metals and dental caries in children, albeit with mixed results [15, 16, 42]. One study reported a positive correlation between tooth decay in children and salivary copper and zinc [15], although another study observed a negative relationship between dental caries and salivary copper [16]. Other studies showed no associations with dental caries and salivary zinc, iron, or manganese [16, 42]. Dietary supplements may also be a source of trace metals in children. Importantly, mixed messaging over dietary supplements could lead to overuse of supplements resulting in high levels in the body [43]. One drawback of the current study is the lack of state-specific environmental exposure data (i.e., soil metal levels, presence of metal industries near residence, air quality data, etc.) and lifestyle factor data, specifically nutritional, dental, and dietary information, which could allow for significant confounding factors to account for the significant associations detected.

Although our study cohort consisted of a large Black adolescent population, the lack of a multiethnic study population limits the generalizability of the presented results. Given the above discussion of the influence of race-ethnicity on various neurophysiological studies of a similar nature, our study’s findings may be specific to the racial composition of our cohort. Therefore, caution should be exercised when extrapolating our results to populations with different racial compositions, specifically when considering the applicability of metal exposure in estimating ADHD risk among adolescents. Future research efforts should build on the results of this study, which highlight saliva as a useful biofluid for environmental exposure assessments in minority adolescent populations in the United States, by including more diverse racial and ethnic populations when investigating ADHD risk factors in adolescents.

In summary, this is the first study to examine associations between levels of metals measured in saliva samples and ADHD outcomes. This study is also strengthened by the consideration of ADHD by diagnostic subtypes, in particular the ADHD-C subtype which includes adolescents exhibiting both hyperactive and inattentive symptoms. Nonetheless, more prospective studies are needed with larger sample sizes to determine the direction and temporality of metal exposure and ADHD development, as well as to examine how exposure to these metals might impact ADHD risk in other racial populations. Such large-scale prospective studies should also include a variety of dietary, nutritional, and environmental exposure variables to better control for the various confounding factors that might drive vulnerability of ADHD burden after contact with these trace elements at levels abnormal for the human body.

## Supplementary Information

Below is the link to the electronic supplementary material.

Supplementary file1 (DOCX 15 KB)

## Data Availability

The data that support the findings of this study are not openly available due to reasons of sensitivity and are available from the corresponding author upon reasonable request. Data are located in controlled access data storage at the University of California, Irvine.

## Abbreviations

ADHD: Attention-deficit/hyperactivity disorder
ADHD-I: ADHD inattentive subtype
ADHD-H: ADHD hyperactive/impulsive subtype
ADHD-C: ADHD combined subtype
DSM-IV: Diagnostic and Statistical Manual of Mental Disorders Fourth Edition
