# Supplementary material for: Increased risk of attention-deficit/hyperactivity disorder in adolescents with high salivary levels of copper, manganese, and zinc
Source: Eur Child Adolesc Psychiatry. 2024 Feb 14;33(9):3091–9. doi: 10.1007/s00787-024-02381-2 (PMC11424719; doi:10.1007/s00787-024-02381-2)
Supplement: Supplementary file 1 — Supplementary file1 (DOCX 15 KB) [file 787_2024_2381_MOESM1_ESM.docx]

**Supplementary Table 1** Quartile ranges of salivary metals levels
